# Pulmonary alveolar microlithiasis combined with gastric mucosal calcification: a case report

**DOI:** 10.3389/fmed.2024.1357260

**Published:** 2024-05-09

**Authors:** Wen-Zhuo Li, Shuo Liu, Ji-Li Luo, Jing Xia

**Affiliations:** ^1^Yunnan Cancer Hospital, Kunming, China; ^2^The First Affiliated Hospital of Kunming Medical University, Kunming, Yunnan, China

**Keywords:** pulmonary alveolar microlithiasis, PAM, gastric mucosal calcinosis, case report, *FBN1*

## Abstract

**Background:**

Pulmonary alveolar microlithiasis (PAM) is a rare disease whose clinical and imaging manifestations are non-specific, characterized by the deposition of microliths, which primarily consist of calcium and phosphorus, within the alveoli. In the cases of PAM, patients combined with calcification of other organs such as gastric mucosal calcification are less common.

**Case presentation:**

A 59-year-old woman was admitted to our hospital due to cough producing white, foamy sputum, accompanied by dyspnea and fever for 20 days. The CT scan showed diffuse ground-glass opacities and calcification of the gastric mucosa. Lung tissue biopsy revealed the presence of calcification and granulomatous foreign bodies in the interstitium and alveolar cavity. In the later stages, she developed painful skin petechiae. For this patient, the diagnosis of PAM, gastric mucosal calcification, and purpura fulminans was made. However, the genetic test results hinted that the patient and her son had a heterozygous mutation in the *FBN1* gene, but her daughter's genetic test results were normal. Although the patient received anti-infection treatment, steroids, and oxygen therapy, her condition did not improve.

**Conclusion:**

We reported a rare case of PAM combined with calcification of other organs and purpura fulminans. Treatment of steroids did not show any benefit. The causative mechanism and effective treatment of this disease remain unclear. More treatments need to be explored.

## Background

Pulmonary alveolar microlithiasis (PAM) is an unusual disease that leads to the accumulation of calcium phosphate microliths throughout the interstitium and alveolar cavity (1, 2). The prevalence of PAM is likely <1 per million. PAM may have no obvious symptoms in its early stages and is detected incidentally by lung imaging. As the lung condition progresses, non-specific manifestations such as cough, dyspnea, and hemoptysis may develop. In a few cases, extrapulmonary organs are involved, with the genitalia being more common, along with other organs such as the kidneys, heart valves, and gallbladder (3, 4). The differentiation of PAM from other calcifying diseases is rather complex. Currently, no definite and effective treatment plan for PAM exists, and most treatment approaches are supportive.

Here, we report the case of a 59-year-old woman diagnosed with PAM using lung tissue biopsy and presented with calcification of gastric mucosa and painful purpura fulminans.

## Case presentation

**Figure 1:**
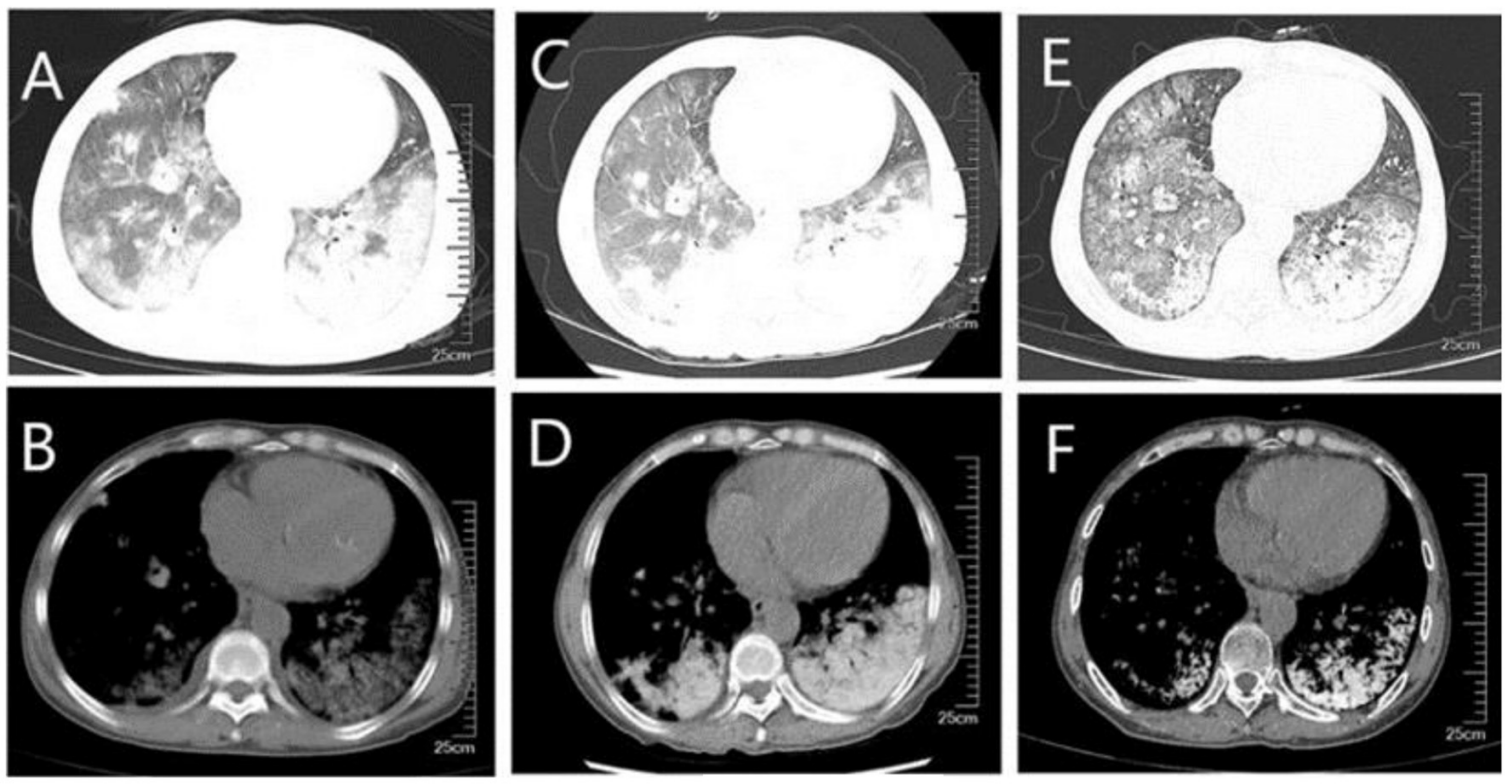
CT of the lungs **(A, B)** diffuse ground-glass shadow and high-density shadow in both lungs were seen in the lung window and mediastinal window when the patient was first admitted to the hospital; **(C, D)** higher density was seen on repeat CT after a period of treatment; **(E, F)** higher density shadow was still seen on repeat CT before discharge, although the ground-glass shadow was absorbed.

**Figure 2:**
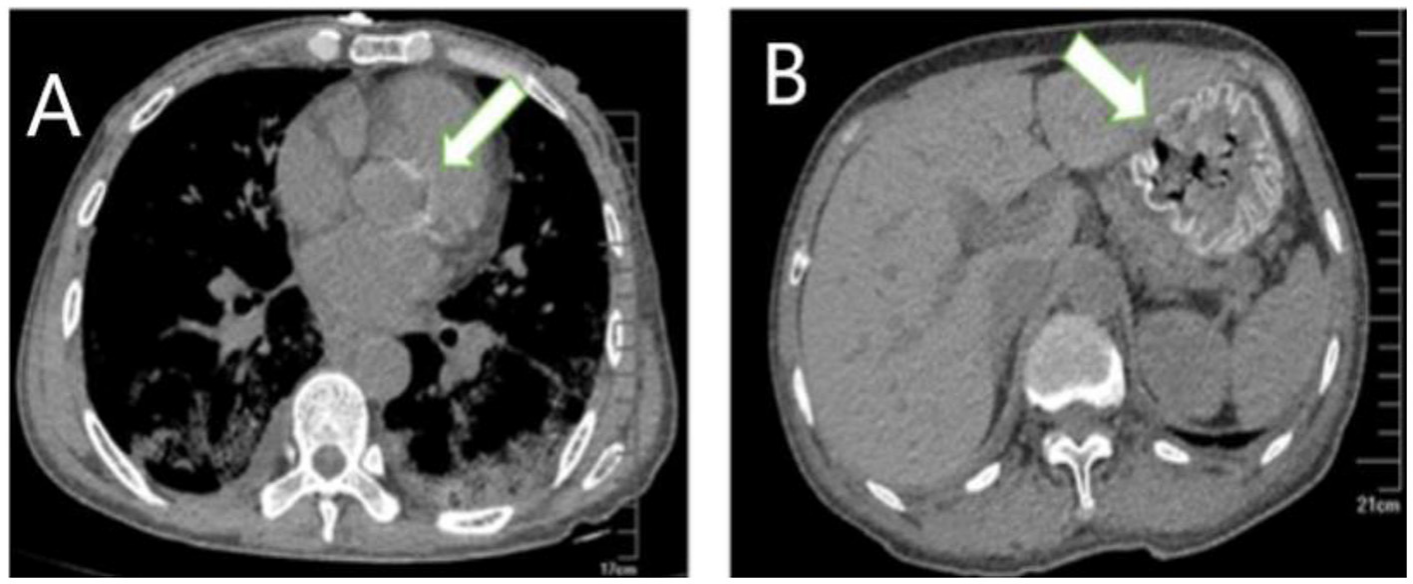
Extrapulmonary organ calcification **(A)** cardiac valve calcification; **(B)** Gastric mucosal calcification.

**Figure 3:**
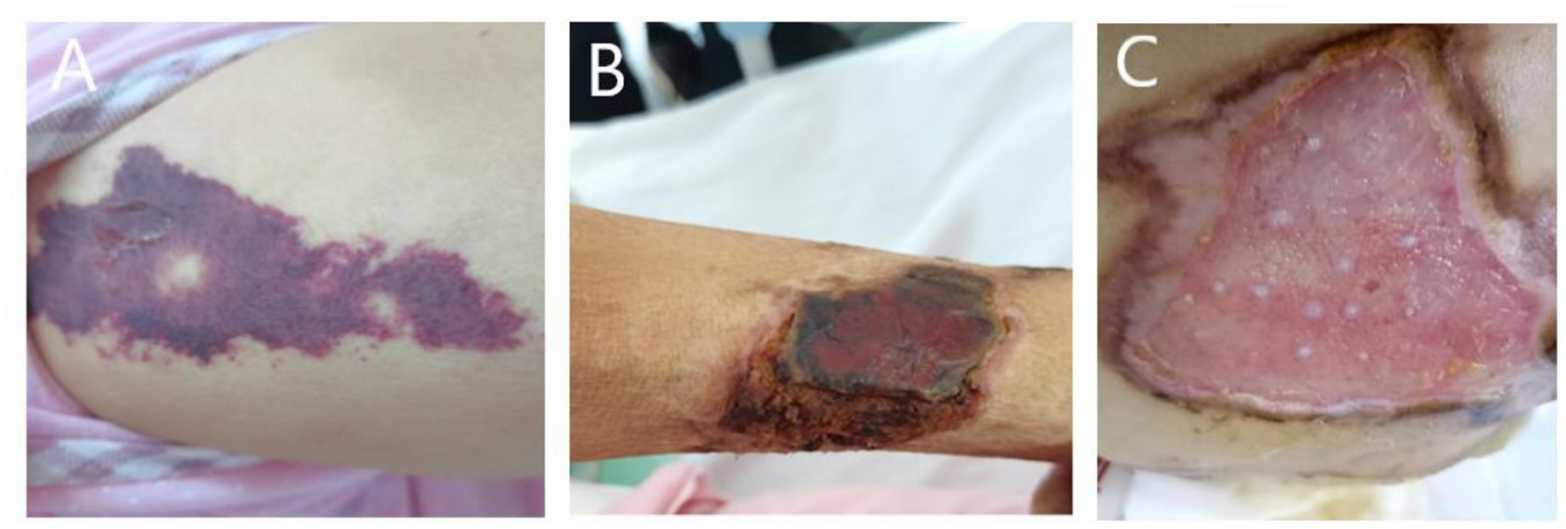
**(A)** Painful skin followed by herpes and purpura; **(B)** follow-up skin condition. **(C)** Patient's buttocks after skin grafting.

**Figure 4:**
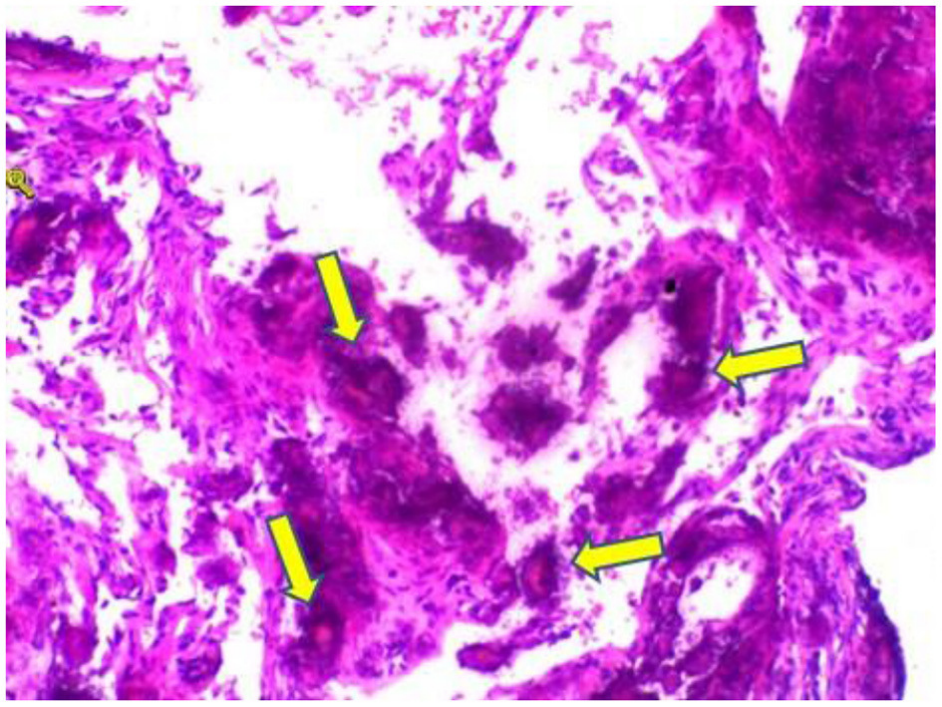
Hematoxylin and eosin stain sections of lung tissue biopsy showing crystalline materials with calcification and foreign body (arrows) granulomas in the interstitium and alveolar cavity (× 100).

A 59-year-old woman was admitted to our hospital on June 2021 because of cough producing white, foamy sputum, accompanied by dyspnea and fever for 20 days. The local hospital considered interstitial pneumonia, and the symptoms did not resolve even after an antiinfective treatment with cefoperazone sodium sulbactam and meropenem for 2 weeks, so she was transferred to our hospital. Lung auscultation showed rales in both lungs. The patient's body temperature rose to 38.5°C. Computed tomography (CT) scan revealed severe diffuse ground-glass opacities (Figure 1), calcification of heart valves (Figure 2), and gastric mucosa (Figure 2). The patient's procalcitonin (PCT) and C-reactive protein (CRP) levels were within the normal range. Based on the patient's symptoms, serological indicators, and imaging presentation, a viral or atypical pathogenic infection was considered. Therefore, she received empirical antiinfective treatment with ganciclovir, moxifloxacin, sulfamethoxazole, and caspofungin. Other treatments included oxygen therapy combined with the prone position and steroids. Her body temperature fluctuated between 37°C- and 37.5°C after 7 days of treatment. However, she still complained of dyspnea and skin pain, followed by the appearance of herpes and petechiae (Figure 3). The CT scan of her lungs did not improve and showed a high-density shadow resembling calcification. To make a definitive diagnosis, we improved relevant inspections. Lung tissue biopsy showed the presence of crystalline materials with calcification and granulomatous foreign bodies in the interstitium and alveolar cavity (Figure 4). Skin biopsy showed purpura fulminans.

Considering that organ calcification may be related to tumors, organ transplantation, renal failure, hyperparathyroidism, abnormal calcium and phosphorus metabolism, and so on, we conducted an etiological search. She did not have a history of organ transplantation, gastric tumor, hypervitaminosis A, or the use of sucralfate. Her creatinine was 75.5 umol/L and her urine output was normal, so renal failure was ruled out. The patient's blood calcium ion level was 2.3 mmoml/L, and blood phosphorus ion level was 1.2 mmol/L, which were within the normal range, so parathyroid disease was not considered. Magnetic resonance imaging (MRI) did not show osteoporosis. Her parents were not consanguineously married, and her siblings had no similar symptoms. However, she said that she had a history of transient elevated calcium levels, the details of which were not clear, which might be a clue.

**Figure 5:**
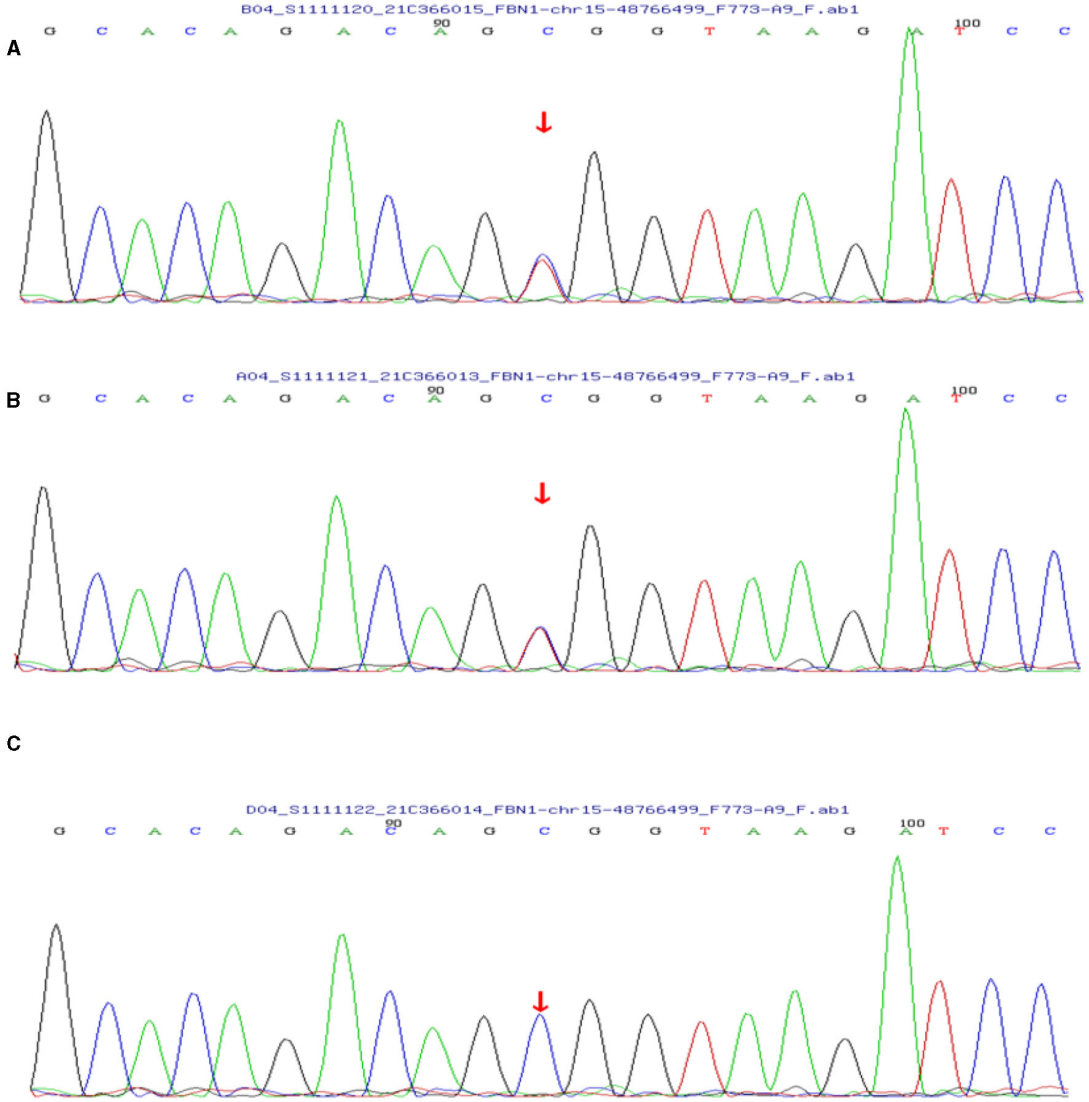
**(A)** The patient's genetic test results suggested a mutation in the *FBN1* gene. **(B)** Her son's test results also showed a mutation in the *FBN1* gene. **(C)** Her daughter's results were normal.

Based on her symptoms and test results, she showed strong indications for PAM, gastric mucosal calcinosis (GMC), and purpura fulminans. Since the disease was associated with a genetic mutation for which there was no effective treatment, we recommended her to undergo genetic testing. However, the genetic test results showed that the patient and her son had a heterozygous mutation in the *FBN1* gene, and sequence 4,163 of the cDNA in the coding region of exon 34 on chromosome 15 was changed from C base to T, thus changing the amino acid GCG (arginine R) to GTG (histidine H) (Figure 5), while her daughter's results were normal. Her 29-year-old son did not show clinical symptoms such as cough or dyspnea, and a CT scan of the lungs did not reveal any abnormalities. The patient did not receive specific medicine interventions (i.e., etidronate, sodium thiosulfate) and then chose to be discharged. During follow-up, the patient showed no improvement in lung imaging and developed hypocalcemia. A partial debridement of the patient's necrotic skin was performed, followed by a skin grafting procedure (Figure 3C).

## Discussion

Calcium deposition of organs and tissue may be metastatic, dystrophic, iatrogenic, and idiopathic in etiology (3, 5). Metastatic calcification is the most frequent subtype in which calcification in normal tissues is caused by hypercalcemia or hyperphosphatemia. Dystrophic calcification occurs as calcium deposition in fibrotic or inflamed tissue without abnormal metabolism of calcium and phosphorus. Iatrogenic calcification is a direct result of pharmacological or therapeutic interventions. Idiopathic calcification occurs in normal tissue without serum biochemical abnormalities. The kidneys and lungs are the preferred sites of calcification, mainly due to the relative intracellular alkalinity (6). Calcinosis rarely occurs in the stomach (GMC) (5). However, our patient did not have a clear history of the abovementioned diseases that could have caused organ calcification.

PAM is a rare lung disease usually caused by a mutation in the *SLC*34A2 gene encoding type IIb sodium phosphate cotransporter called NPT2b (2). There is no literature suggesting that the disease is caused by mutations in other genes (4). However, in this case, the patient had a mutation in the *FBN1* gene. The primary function of the *FBN1* gene is to encode fibrillin-1, which causes it to polymerize into microfibrils in connective tissue, forming the tissue-specific structural framework (7). The mutation was reported more frequently in patients with Marfan syndrome (MFS) (8), which is a relatively common genetic disorder of connective tissue (1 in 3,000–5,000) with different clinical features in the musculoskeletal, cardiovascular, and ocular systems (7). Calcification in patients with MFS usually occurs in connective tissues such as the aorta and heart valves, but alveolar calcification has not been reported (9). The patient and her family did not have symptoms associated with MFS but rather presented with multiorgan calcification as well as skin lesions, which were rarely reported. The two major building blocks of fibrillin-1 are the calcium-binding epidermal growth factor-like domain (cbEGF) and the transforming growth factor-binding protein-like domain (TB or 8-Cys) (10). Most mutations reported to date affect residues of the calcium-binding common sequence, resulting in an incomplete calcium-binding protein structural domain and reduced calcium-binding capacity of cbEGF (11). Although organ calcium deposition caused by mutations in the *FBN1* gene has been rarely reported, this mutation may be a potential mechanism for the development of multiorgan calcification in this patient.

Most patients with PAM are asymptomatic at diagnosis. As the disease progresses, dyspnea often develops along with cough, chest pain, hemoptysis, and pneumothorax, which are non-specific. Over time, adult patients often experience deterioration in lung function, and respiratory failure due to chronic cor pulmonale is the leading cause of death (12). In this case, the patient's main symptoms were cough and dyspnea with nasal cannula oxygenation, and oxygen saturation was maintained at 92%. No changes in cardiac structure or function were noted, so she was still in the early stages of the disease. The diagnosis of PAM is usually confirmed by radiological and histopathological features. High-resolution computed tomography typically shows diffuse hyperdense micronodules, which are most widely located in the middle and lower lung lobes just like this patient (10). In some cases, the calcified areas of the lungs are more extensive, which is called a sandstorm-like pattern, and even a black pleural line will appear between the lung parenchyma and the ribs (12, 13). In addition, calcification of extrapulmonary tissues, such as gastric mucosal and cardiac valve, was observed in this case, which was not reported in other cases. Microliths visible in alveolar lavage fluid or lung biopsy are necessary for a definitive diagnosis, while detection of genetic mutations is not necessary for the diagnosis. Treatments reported in the past included bisphosphonates, corticosteroids, sodium thiosulfate, and low-phosphorus diets, but none have been reported to be effective. Lung transplantation in advanced stages of PAM may be a potential treatment.

In this case, we suggest that PAM belongs in metastatic calcification or idiopathic calcification. Although calcium and phosphorus levels were normal upon admission, the patient had a history of transiently elevated calcium levels during follow-ups, which was not taken seriously and left untreated. Iatrogenic calcification and dystrophic calcification were excluded because she did not use pharmacological or therapeutic interventions of calcium, and antibacterial drugs did not reduce symptoms or improve imaging performance. Corticosteroid therapy did not affect this case. It is also not known how the patient's electrolyte levels will change as the disease progresses.

## Conclusion

PAM is already a rare disease, and there is even less literature on the combination of GMC and skin lesions. The causative mechanism of PAM remains unclear, and it may be a common manifestation of multiple diseases. At present, no effective medical therapy for PAM exists; nevertheless, lung transplantation can be an effective treatment option for end-stage patients with grave respiratory deficiency. More studies are needed to help understand the risk factors, pathogenic mechanism, treatment methods, and outcome of PAM.

## Data availability statement

The original contributions presented in the study are included in the article/supplementary material, further inquiries can be directed to the corresponding author.

## Ethics statement

Ethical approval was not required for the studies involving humans because this study is a case report, a single case observational study, and the informed consent of the patient was obtained. The studies were conducted in accordance with the local legislation and institutional requirements. The human samples used in this study were acquired from a by-product of routine care or industry. Written informed consent to participate in this study was not required from the participants or the participants' legal guardians/next of kin in accordance with the national legislation and the institutional requirements. Written informed consent was obtained from the individual(s) for the publication of any potentially identifiable images or data included in this article.

## Author contributions

W-ZL: Writing – original draft, Writing – review & editing. SL: Methodology, Writing – review & editing. J-LL: Investigation, Writing – review & editing. JX: Investigation, Writing – review & editing.
